# Tunable Goos–Hänchen Shift in Symmetric Graphene-Integrated Bragg Gratings

**DOI:** 10.3390/mi16101184

**Published:** 2025-10-20

**Authors:** Quankun Zhang, Miaomiao Zhao, Hao Ni, Hao Wu, Fangmei Liu, Fanghua Liu, Zhongli Qin, Dong Zhong, Zhe Liu, Xiaoling Chen, Dong Zhao

**Affiliations:** 1 School of Electronic and Information Engineering, Hubei University of Science and Technology, Xianning 437100, China; zhangquankun@hbust.edu.cn (Q.Z.);; 2 Hubei Provincial Key Laboratory of Optoelectronic Information and Intelligent Control, Hubei University of Science and Technology, Xianning 437100, China

**Keywords:** Goos-Hänchen shift, graphene arrays, Bragg grating, resonant cavity

## Abstract

We theoretically analyze the spatial Goos-Hänchen (GH) shifts in symmetric Graphene-Integrated Bragg Gratings (GIBGs), where monolayer graphene arrays act as tunable input/output couplers, and a periodically inserted dielectric layer forms a resonant cavity. By optimizing the cavity design, we achieve a GH shift of 1766λ, surpassing the conventional limit of hundreds of wavelengths under single-parameter tuning. The direction and magnitude can be actively controlled by the graphene’s chemical potential, grating geometry, or dielectric thickness. This mechanism may enable high-sensitivity refractive index sensors or adaptive optical devices.

## 1. Introduction

When a finite-sized wavefront beam undergoes reflection or refraction at an interface, its propagation path exhibits deviations from geometric optics predictions, manifesting as the lateral Goos-Hänchen (GH) shift [1,2]. This phenomenon arises from phase delay variations across the constituent plane wave components, with the displacement magnitude proportional to the angular derivative of the reflection coefficient’s phase [3]. The exceptional phase sensitivity of GH shifts has enabled diverse applications including subwavelength-resolution optical sensing [4,5], wavelength-selective photonic systems [6,7], nonlinear optical switching [8,9], and chip-scale photonic devices [10]. Furthermore, by introducing materials that are sensitive to electric fields, magnetic fields, or temperature, dynamic control of the GH shift can be achieved: for example, tuning the GH shift in a metal–insulator–semiconductor structure using an external bias [11]. Modulating the GH shift of the reflected beam at the surface of a colloidal ferrofluid using an external magnetic field [12]. Temperature-dependent GH shifts have been quantitatively characterized for aluminum surfaces in terahertz regimes [13], and enhanced thermal sensitivity has been demonstrated through graphene-BK7 substrate hybrids [14]. This multidimensional field-responsiveness significantly expands GH shift engineering capabilities for adaptive photonic systems and multifunctional optical sensors.

The honeycomb lattice of graphene gives it linear Dirac dispersion and electrically tunable optical conductivity [15]. By adjusting the Fermi level through gate voltage or chemical doping, its surface plasmon polariton (SPP) dispersion can be flexibly changed, thereby significantly modulating the reflection phase slope, providing a new idea for amplifying the GH shift [16,17]. Previous studies have achieved GH shifts of hundreds to thousands of wavelengths in single-layer graphene–metal composite films [18], graphene–photonics crystal heterostructures [19], and grating waveguides containing graphene [20]. These systems often require a trade-off between displacement amplitude, spectral tunability, and structural robustness.

Bragg gratings, due to their wavelength selectivity and light field control capabilities, are widely used in fiber-optic sensing, optical communication, and integrated photonics [21,22]. Traditional Bragg gratings are mostly made of silicon-based or polymer materials, with limited sensitivity, tunability, and compatibility with new nanomaterials [23]. Graphene, with its excellent electrical, optical properties, and atomic thickness, has become an ideal candidate for photonic device design [24,25]. Its symmetric band structure (conduction and valence bands symmetrically distributed around the Fermi level) further enhances its modulation potential in light–matter interactions [26]. When graphene is periodically arranged in the transverse direction, it can form photonic crystals with photonic band gaps in the wave vector space [27]. Compared with single-layer graphene, these periodic structures exhibit stronger optical resonance, steeper band edges, and superior extinction performance [28,29]; moreover, the band gap width can be flexibly changed through chemical potential regulation, further enhancing the band gap control ability [30].

Inspired by these advances, this paper investigates the GH shift in a Bragg grating structure composed of symmetrically arranged graphene arrays. By optimizing the layer spacing and grating period, a significant displacement of up to 1766λ is achieved. Subsequently, by tuning the incident angle, graphene relaxation time, graphene chemical potential, and dielectric layer refractive index, the flexible control of the GH shift by each parameter is demonstrated. These results provide a reference for the design of high-sensitivity optical sensors.

## 2. Structure and Method

**Figure 1:**
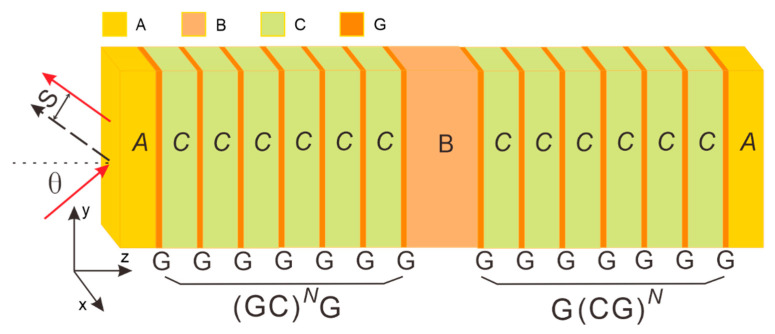
Schematic diagram of the proposed graphene-integrated Bragg gratings (GIBGs).

A schematic of the proposed stack is shown in Figure 1 and can be compactly written as $A{\left(GC\right)}^{N}GBG{\left(CG\right)}^{N}A$. In this notation, *A*, *B*, and *C* label three distinct SiO_2_ dielectric regions, *G* stands for monolayer graphene, and *N* is the number of periods in each Bragg grating. The device contains two identical gratings, two *A*-type spacers, and one *B*-type spacer. Each grating is formed by alternating graphene sheets and *C*-type dielectrics, with graphene acting as both the entrance and exit boundary. The thicknesses of layers *A*, *B*, and *C* are 4 µm, 4 µm, and 100 nm, respectively, while the graphene monolayer is 0.34 nm thick. Illumination is assumed to be transverse-magnetic (TM) polarized light; θ indicates the angle of incidence and *S* denotes the resulting Goos-Hänchen shift.

In this section, we combine the transfer matrix method (TMM) with the phase method [31] to calculate the reflectance, reflection phase, and GH shift in the structure. The refractive index of each layer can be obtained by $n_{j}=\sqrt{\varepsilon_{j}}$, where the subscript *j* denotes the layers *A*, *B*, *C* and *G* in the GIBGs structure. According to TMM [31], the characteristic matrix of each layer is:

(1) $$M_{j}=\left[\begin{array}{cc} {\cos q}_{j} & \left(-i{\sin q}_{j}\right)/p_{j}\\ -ip_{j}{\sin q}_{j} & {\cos q}_{j} \end{array}\right]$$

where $p_{j}$ and $q_{j}$ are written as:

(2) $$p_{j}=\frac{\sqrt{{\varepsilon_{j}-n}_{1}^{2}{sin}^{2}\theta_{1}}}{\varepsilon_{j}}$$

(3) $$q_{j}=\frac{2\pi d_{j}}{\lambda }\sqrt{{\varepsilon_{j}-n}_{1}^{2}{sin}^{2}\theta_{1}}$$

where $d_{j}$ represents the thickness of layers *A*, *B*, *C* and *G*. For the GIBGs stack, the overall transfer matrix is obtained by cascading all individual layers, giving M = $\prod_{j=1}^{4N+4}M_{j}$ = [*M*_11_, *M*_12_; *M*_21_, *M*_22_]. Both the entrance and exit media are free space, so the corresponding admittances are set to $\eta_{0}=\eta_{4N+5}={\left(\varepsilon_{0}/\mu_{0}\right)}^{1/2}$. $\mu_{0}$ is the vacuum permeability. The reflection coefficients (*r*) of the GIBGs stack are obtained from:

(4) $$r=\frac{{(M}_{11}+M_{12}\eta_{4N+5})\eta_{0}-{(M}_{21}+M_{22}\eta_{4N+5})}{{(M}_{11}+M_{12}\eta_{4N+5})\eta_{0}+{(M}_{21}+M_{22}\eta_{4N+5})}$$

The reflectance of the structure is given by $R={rr}^{*}$. The complex reflection coefficient can be written in polar form as $r=\left|r\right|exp(i\varphi_{r})$, where $\varphi_{r}$ denotes its phase.

Adopting the stationary-phase approach [32], the lateral GH shift in the reflected beam is found to obey:

(5) $$S=-\frac{d\varphi_{r}}{dk_{y}}.$$

The component of the wave vector oriented in the y-direction is represented by $k_{y}$. When the incident light intensity is weak, the nonlinear effects of graphene can be neglected, and we consider only the linear component of its surface conductivity. This linear component can be estimated using the Kubo formula [33]. The effective dielectric constant of graphene is given by $\varepsilon_{g}=1+i\sigma_{g}\eta_{0}/k_{0}d_{g}$, where $k_{0}=2\pi /\lambda$ is the free-space wave vector, i denotes the imaginary unit, and$\;\sigma_{g}$ is the total surface conductivity of graphene, given by the sum of the intra-band ($\sigma_{\mathrm{intra}}$) and inter-band ($\sigma_{\text{inter}}$) contributions.

The intra-band conductivity is calculated from.

(6) $$\sigma_{\mathrm{intra}}=i\frac{e^{2}k_{B}T_{g}}{\pi \hbar^{2}(w+i/\tau )}\left[\frac{\mu }{k_{B}T_{g}}+2\ln[e^{-\mu /k_{B}T_{g}}+1]\right],$$

where *k_B_* is the Boltzmann’s constant, ℏ is the reduced Planck’s constant. The symbols for other parameters are described in Table 1.

The inter-band conductivity can be simplified as

(7) $$\sigma_{\mathrm{inter}}=i\frac{e^{2}}{4\pi \hbar^{2}}\ln\frac{2\left|\mu \right|-\hbar (\omega +i\tau^{-1})}{2\left|\mu \right|+\hbar (\omega +i\tau^{-1})}.$$

Both inter-band and intra-band transition terms depend on the chemical potential *μ* and the angular frequency *ω* of the incident light; consequently, the surface conductivity of graphene is an explicit function of *μ* and *ω*. Moreover, continuous tuning of the chemical potential is realized by sweeping the externally applied gate voltage: a positive bias shifts *μ* upward, whereas a negative bias shifts it downward. This variation directly modifies the surface conductivity of graphene, enabling flexible electrical control of its optical response via the gate voltage.

## 3. Numerical Results

**Figure 2:**
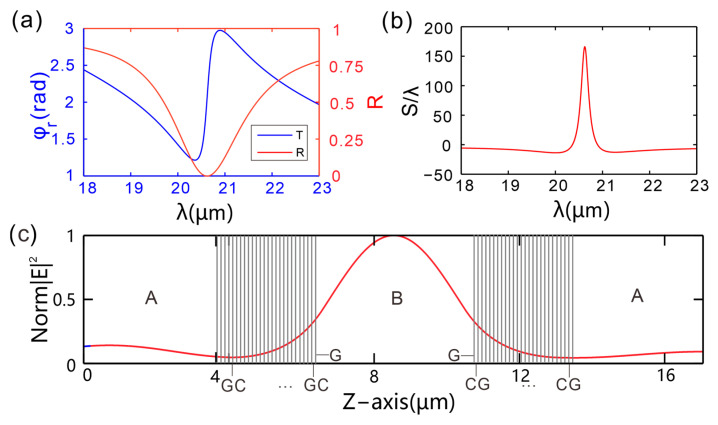
(**a**) Reflectance spectrum and phase of the reflection coefficient as functions of incident wavelength. (**b**) GH shift. (**c**) Magnetic field intensity distribution in the GIBGs structure as a function of distance.

In the numerical calculations, the thicknesses of the first and second dielectric layers are both 4 µm, and the thickness of the third dielectric layer is 100 nm. The values of other parameters are shown in Table 1. First, the GH shift in the GIBGs structure and its corresponding reflectance and reflection phase as functions of wavelength are investigated. Figure 2a shows the corresponding reflectance and reflection phase. It can be seen that the reflection phase of the GIBGs structure decreases with increasing wavelength and then reaches a trough, where the phase curve undergoes a significant abrupt change to a peak. However, as the wavelength continues to increase, the reflection phase of the GIBGs structure gradually decreases. The reflectance curve shows a reflectance drop to zero at a wavelength of 20.58 µm. In Figure 2b, the GH peak of the GIBGs structure reaches 165.93*λ* at the operating wavelength of 20.58 µm. At this exact operating wavelength, the GH peak corresponds to the position where the reflection phase slope is the largest. The abrupt change in the reflection phase leads to a sharp increase in the magnitude of the GH shift.

To elucidate the intrinsic mechanism underlying the significant GH shift in the GIBGs structure, Figure 2c shows the distribution of the electric field intensity modulus as a function of distance under TM wave incidence with an incident wavelength *λ* of 20.58 µm. From left to right in the figure, the gray lines correspond to the edges of the layered materials in the GIBGs structure, consistent with the model in Figure 1. The first gray line is at 4 µm, with its left side representing dielectric layer *A*. The positions of the gray lines coincide with the locations where monolayer graphene is embedded, and the regions between the gray lines are dielectric layer *C*. The middle part is cavity *B*. The right side of the last gray line represents the rightmost dielectric layer *A*. The red curve depicts the distribution of the electric field intensity modulus. As can be seen from the figure, the electric field intensity of the GIBGs structure varies along the *Z*-axis, with most of the energy tightly confined in the middle layer. The electric field is strongest at the center of the middle cavity and decays as it extends outward from the center. The sharp electric field peak in region *B* in Figure 2c directly indicates that the graphene-integrated Bragg grating strongly couples with the cavity in region *B* at λ = 20.58 µm, forming a resonant state. This resonance corresponds to a sharp reflection valley in the reflection spectrum and is accompanied by a drastic change in the reflection phase with the incident Angle (phase leap), which is completely consistent with the trend of the red solid line in Figure 2a. It is precisely this steep phase change that directly gives rise to the huge GH shift shown in Figure 2b.

**Figure 3:**
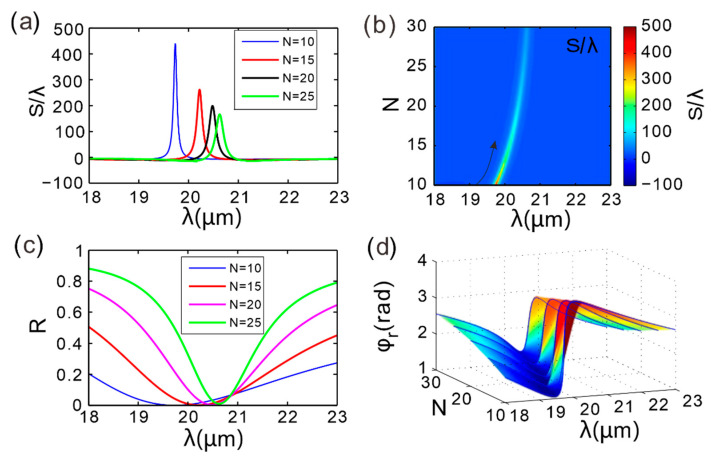
(**a**) GH shift in the reflected light from the GIBGs structure as a function of wavelength for different periods *N*. (**b**) Dependence of GH shift on incident wavelength and *N*. The parameter space is composed of incident wavelength and period number. (**c**) Variation in reflectance with incident wavelength. (**d**) Reflection phase, with the parameter space composed of incident wavelength and period number.

Figure 3 illustrates the variations in the GH shift, reflectance, and reflection phase of the reflected light from the composite structure at different spatial periods *N*, with the incident wavelength ranging from 18 µm to 23 µm. The blue, red, black, and green solid lines in the figure correspond to the calculated results of the structure for spatial periods *N* of 10, 15, 20, and 25, respectively. As shown in Figure 3a, one distinct GH-shift maximum appears across the examined wavelength span when the grating period number is set to *N* = 10, with a magnitude of approximately 439λ, occurring at a wavelength of 19.73 µm. As *N* increases to 15, 20, and 25, the peak magnitudes of the GH shift are approximately 262λ, 199λ, and 166λ, respectively, with corresponding peak positions at wavelengths of 20.22 µm, 20.48 µm, and 20.63 µm. As *N* increases from 10 to 25, the position of the GH peak gradually shifts to longer wavelengths, maintaining a single peak throughout. It is evident that the GH shift value in this structure varies with the period number, indicating that the period number can be effectively tuned to achieve desired outcomes.

To more clearly observe the changes in the GH peak position as *N* varies from 10 to 25, Figure 3b plots the GH shift as a function of *N* and wavelength. It is found that the period number can alter the magnitude of the GH peak. Specifically, the GH shift value changes significantly as *N* increases from 10 to 15 (black arrow), and changes more slowly as *N* increases from 15 to 25. Figure 3c reveals that the reflectance minima align precisely with the GH-shift maxima depicted in Figure 3a.

Figure 3d shows the reflection coefficient phase in the parameter space composed of Bragg grating period number and incident wavelength. As the number of periods *N* increases, the slope of the reflection phase gradually flattens: the slope at *N* = 10 is steeper than that at *N* = 15, and the slope further decreases when *N* increases from 20 to 30. This trend directly explains the phenomenon in Figure 3a that the peak value of GH displacement decreases as *N* increases.

By optimizing the period number (*N* =10 to *N* = 15), the GH shift amplitude can be tuned from 166λ to 439λ while maintaining a stable operating wavelength band (20 ± 0.5 µm). At *N* = 15, the displacement retains 60% of the initial value, significantly reducing the complexity of fabrication. This provides a key design criterion for balancing high performance and low cost.

**Figure 4:**
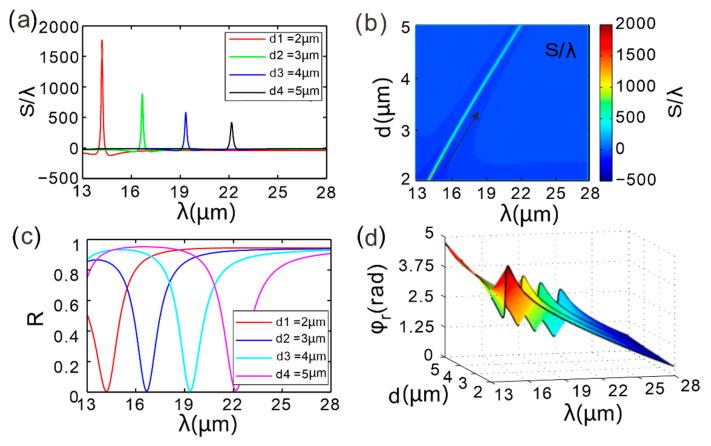
(**a**) GH shift as a function of wavelength for different thicknesses of dielectric layer *B*. (**b**) Dependence of GH shift on incident wavelength and thickness of dielectric layer *B*. (**c**) Variation in reflectance with incident wavelength. (**d**) Reflection phase. The parameter space is defined by incident wavelength and thickness of dielectric layer *B*.

To understand the role of thickness regulation in enhancing the GH shift, Figure 4a displays GH-shift spectra obtained by systematically sweeping the thickness of dielectric layer *B* through 2 µm, 3 µm, 4 µm, and 5 µm. It can be seen that within the wavelength range of 13–28 µm, each curve exhibits only one GH shift. The peak value of the GH shift decreases with increasing thickness of dielectric layer *B*, and the peak position shifts towards longer wavelengths. Figure 4b shows the variation in the GH shift with the thickness of the middle dielectric layer and wavelength in the designed structure. It can be observed that there is a slanted line in the GH shift spectrum (black arrow), with colors and positions changing with wavelength. Specifically, within the thickness range of 2–3 µm, the amplitude of the GH shift peak rapidly decreases with increasing thickness, while within the thickness range of 4–5 µm, there is almost no change in color. The overall peak position also shifts towards longer wavelengths. Figure 4c shows the reflectance as a function of wavelength. The reflectance of the GIBGs structure remains high over most of the wavelength range, with the reflectance dip occurring at the same position as the GH peak in Figure 4a. The spectral location of peak absorption is highly sensitive to the dielectric-layer thickness. Figure 4d presents the phase of the reflection coefficient mapped across a parameter space spanned by the thickness of the second dielectric layer and the incident wavelength. As can be seen from Figure 4d, as the thickness *d* of the intermediate medium layer increases, the reflection phase changes more gently with the wavelength. By comparing with Figure 4a, it can be known that when *d* = 2 µm, the peak of GH displacement occurs at 14.19 µm, with an amplitude of 1766λ. When *d* = 5 µm, the peak shifts to 22.16 µm and the amplitude is only 1/4.2 of the former. The fundamental reason lies in that the increase in thickness reduces the absolute value of the reflection phase slope, resulting in a significant decrease in the GH enhancement factor (proportional to the phase slope).

By precisely controlling the thickness of dielectric layer *B* (within the range of 2–5 µm), dual-tuning of GH shift in terms of both intensity and wavelength can be achieved in the 13–28 µm wavelength band: a thin layer (2 µm) provides ultra-high displacement sensitivity (1766λ), and increasing the thickness to 5 µm can extend the operating wavelength to 22 µm (with the displacement maintained at >400λ). This design offers the simplest control paradigm for wavelength-selectable giant displacement devices, avoiding complex parameter optimization and significantly enhancing the engineering feasibility of the device.

**Figure 5:**
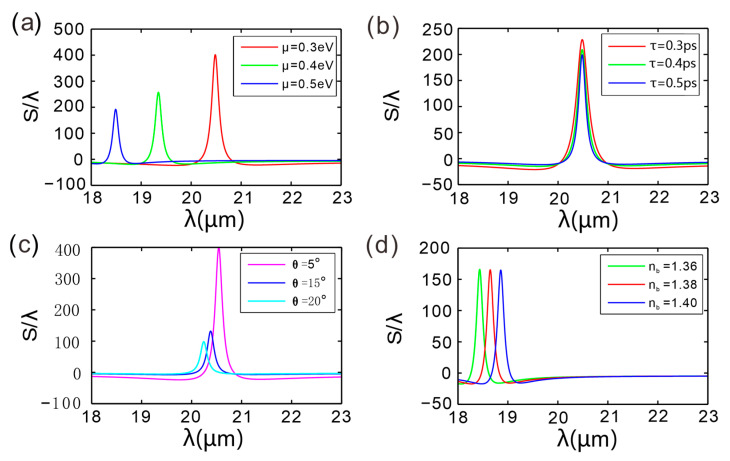
The GH shift as a function of incident wavelength for the GIBGs structure under different (**a**) chemical potentials, (**b**) relaxation times, (**c**) incident angles, and (**d**) refractive indices of dielectric layer *B*.

Figure 5a illustrates how the GH shift in the GIBGs device evolves with wavelength when the graphene chemical potential is tuned to 0.3 eV, 0.4 eV, and 0.5 eV, revealing that the overall trend of the GH shift is consistent across the three chemical potentials: a peak appears for each grating period. As the chemical potential decreases, the peak redshifts towards longer wavelengths and its amplitude increases. From Figure 5b, it can be seen that the overall trend of the GH shift spectrum of the GIBGs structure with wavelength is similar for different relaxation times, but the GH peak value decreases with increasing relaxation time, while the peak position remains almost unchanged. Figure 5c shows the GH shift in the GIBGs structure as a function of wavelength for different incident angles: the GH peak value significantly increases as the incident angle decreases. Specifically, at *θ* = 20° and 15°, the GH peak values are 98 and 132 times the incident wavelength, respectively; when *θ* is further reduced to 5°, the peak jumps to 398 times. Figure 5d demonstrates the influence of the dielectric-layer refractive index on the GH shift. Incrementing the index in 0.02 steps leaves the peak amplitude nearly constant while shifting its position markedly toward longer wavelengths. Due to the change in refractive index, the reflection coefficient will change, causing the position of the resonance mode to shift.

The reduction in the chemical potential of graphene, for instance, from 0.5 eV to 0.3 eV, significantly increases the GH shift peak and shifts its position towards longer wavelengths. Lowering the Fermi level enhances the photon–electron coupling efficiency, thereby amplifying the GH effect. The GH shift peak monotonically decays with increasing relaxation time (carrier mobility improvement), while the peak wavelength remains essentially unchanged. Carrier transport properties mainly affect the magnitude of the shift rather than the resonance position, with high mobility actually weakening the localized field enhancement effect. When the incident angle decreases from 20° to 5°, the peak value of the GH shift dramatically increases, quadrupling the original value and showing an exponential growth trend, indicating that a significant GH shift can be obtained under conditions of small-angle incidence. For every 0.02 increase in the refractive index of dielectric layer *B*, the GH peak position redshifts by about 0.5 µm, while the amplitude change is less than 3%. The refractive index primarily regulates the resonance wavelength, providing a fine-tuning mechanism for the device’s operating wavelength band and having a negligible impact on the stability of the shift. This multi-parameter tunability offers a flexible design space for graphene-based optical displacement devices.

**Figure 6:**
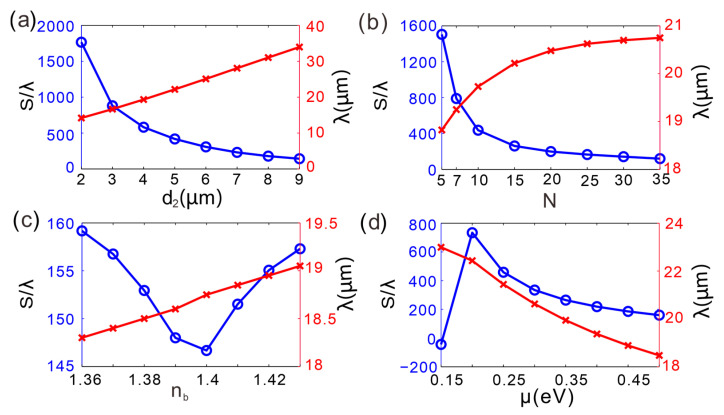
Variation in the GH shift peak and incident wavelength with (**a**) thickness of dielectric layer *B*, (**b**) Bragg grating period number, (**c**) refractive index of dielectric layer *B*, and (**d**) chemical potential in the GIBGs structure.

Figure 6a maps the wavelength-dependent GH-shift resonance of the GIBGs heterostructure while stepping the thickness of dielectric layer *B* through discrete values. As the thickness of the dielectric layer *B* increases from 2 µm to 9 µm, the GH shift peak rapidly decreases and then levels off. At $d_{2}$ = 2 µm, the GH shift peak is close to 1766 times the incident wavelength; when $d_{2}$ increases to about 5 µm, the GH shift peak drops to around 417 times the incident wavelength and continues to decrease slowly thereafter. The red curve on the right, representing the incident wavelength, gradually increases with the increase in dielectric layer thickness $d_{2}$, rising slowly from about 15 µm to around 35 µm. The increase in dielectric layer thickness leads to a decrease in the GH shift peak, and the incident wavelength correspondingly increases. Evidently, the dielectric-layer thickness governs not only the magnitude of the GH shift but also the corresponding incident wavelength.

Figure 6b displays the GH-shift peak versus the incident wavelength for different Bragg grating periods *N*. As *N* grows from 5 to 35, the peak first plunges from nearly 1505λ to 1200λ and then saturates. The resonance wavelength increases rapidly for small *N* and then stabilizes around 18–21 µm. Hence, adding more periods primarily damps the peak amplitude without strongly affecting the wavelength.

Figure 6c presents the GH-shift peak as a function of the refractive index of dielectric layer *B*. When $n_{b}$ is raised from 1.36 to 1.42, the peak first decreases from 160λ to 145λ and then slightly rebounds. The resonance wavelength red-shifts monotonically from 18 µm to 19.5 µm, indicating that $n_{b}$ provides fine control over both amplitude and spectral position.

Figure 6d illustrates the dependence on graphene chemical potential *μ*. Increasing *μ* from 0.2 eV to 0.5 eV monotonically reduces the peak from 734*λ* to 160*λ*, while the resonance wavelength red-shifts from 18 µm to 23 µm. Consequently, *μ* offers an effective knob for simultaneously tuning the GH-shift magnitude and the operating wavelength.

Fixing all other parameters at their nominal values, we perform a single-parameter scan (Table 2) that yields the record GH shift of 1766λ when the thickness of dielectric *B* (d_2_) is varied from 2 µm to 6 µm (λ = 13–28 µm). Multi-parameter optimization (*N* = 10, *μ* = 0.2 eV, d_2_ = 4 µm) further pushes the peak beyond 3535 λ, confirming that 1766λ is the controlled-tuning maximum rather than an absolute upper limit. As shown in Table 2 of that reference, a high sensitivity of ≈60λ per 0.1 V gate voltage change was achieved, enabling an electrically controlled GH tuning range of over 500λ. In practical applications, the electro-optical performance of this device is applied to non-mechanical, mid-infrared light attenuators and sensitive voltage displacement sensors. In addition, CVD-grown dielectrics and commercial monolayer graphene are alternately stacked by standard transfer/ALD to form the Bragg superlattice with atomic-scale thickness control [34,35,36].

**Figure 7:**
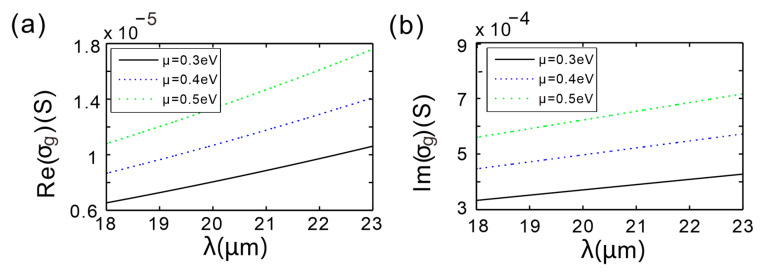
(**a**) Real part of graphene surface conductivity versus incident wavelength for different chemical potentials. (**b**) Imaginary part of graphene surface conductivity versus incident wavelength for different chemical potentials.

Figure 7a shows the real part of the graphene surface conductivity, Re(*σ_g_*), for different chemical potentials. For the three chosen values *μ* = 0.3, 0.4 and 0.5 eV, three Re(*σ_g_*) curves are plotted versus the incident wavelength. At a fixed chemical potential, Re(*σ_g_*) grows as the wavelength increases. Conversely, for a fixed wavelength, a higher chemical potential yields a larger Re(*σ_g_*). Because Re(*σ_g_*) is directly linked to the imaginary part of the graphene equivalent permittivity, Im(*ε_g_*), it quantifies the optical losses in graphene; hence a larger Re(*σ_g_*) implies stronger dissipation in the system. Increased loss flattens the reflection-phase variation around resonance, reduces the absolute slope of the phase, and consequently suppresses the peak GH shift. This corroborates the observation in Figure 5a that *μ* = 0.3 eV produces the largest GH-shift peak.

Figure 7b presents the imaginary part of the surface conductivity, Im(*σ_g_*), as a function of wavelength. For *μ* = 0.3, 0.4 and 0.5 eV, Im(*σ_g_*) rises with increasing wavelength on each curve. Likewise, at any given wavelength, a higher chemical potential gives a larger Im(*σ_g_*). The imaginary part of the conductivity governs the real part of *ε_g_*, and since Im(*σ_g_*) is tunable via the chemical potential *μ*, an external gate voltage can be used to tailor the effective permittivity and thereby control the GH displacement.

**Figure 8:**
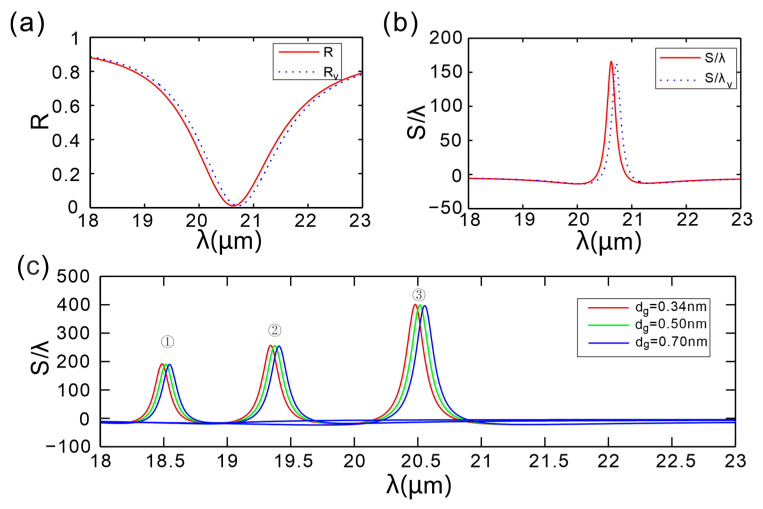
(**a**) Reflection spectra as a function of wavelength for varying dielectric-A thickness. (**b**) GH shift as a function of wavelength for varying dielectric-A thickness. (**c**) GH shift as a function of wavelength for varying graphene thickness.

To assess fabrication robustness, we generate 200 random samples to mimic thickness uncertainty and evaluate the resulting Goos-Hänchen shift distribution. Figure 8 presents a comparison of the reflection spectra and the GH-shift peak results. The solid lines (labeled *R* and *S/*λ) represent the predicted results of the theoretical model, whereas the dashed lines (labeled *R*_v_ and *S/*λ_v_) represent the simulation results after accounting for the irregularities. It can be observed that the data on reflectance align with the theoretical expectations. In these Monte-Carlo trials, a ±3 nm (3*σ*) random fluctuation in the thickness of dielectric A yields a normal distribution of the GH shift with a 3*σ* variation of only 1.2% (166λ → 164λ).

Simultaneously sweeping chemical potential *μ* and single-layer thickness *h*_g_, Figure 8c shows that for the same *μ* the three curves with *h*_g_ = 0.34–0.70 nm almost overlap (①–③), yielding a GH-peak variation < 3%. Along the μ axis, however, GH jumps from 190λ to 400λ. Thus, chemical potential is the dominant lever, while thickness drift introduces only negligible offset, confirming that the device is robust against typical single-layer thickness fluctuations.

Unlike traditional integrated Bragg gratings (IBGs), whose tuning relies on thermal-optical effects (e.g., silicon/SiN heating strips), electro-optical modulation (e.g., P-I-N carrier injection), or phase-change materials (e.g., GST or Sb_2_S_3_ crystallization–amorphization) [37,38,39,40], and whose functions are concentrated on narrowband filtering, wavelength division multiplexing, or phase modulation; Moreover, the dynamic range is usually limited. This work introduces a single-layer graphene strip array into the Bragg mirror period, forming an atomically thin “tunable mirror.” By electrically tuning the chemical potential of graphene via gate voltage, the reflection phase can be continuously controlled, enabling a Goos-Hänchen (GH) shift of up to 1766λ; with multi-parameter collaborative optimization, the peak value can be further increased to 3535λ. Compared with photonic crystals that exploit exceptional points (10^3^λ) [41] or guided-mode resonance gratings (500λ, requiring sub-100 nm slot etching) [42], this approach requires no precise defect engineering or critical alignment, and offers a new functionality—continuously tunable beam shifting—for IBG-based devices.

## 4. Conclusions

In summary, we theoretically investigate the Goos-Hänchen (GH) shift in a Bragg grating structure composed of symmetrically arranged graphene arrays. When TM-polarized light is incident, a significant GH shift can be observed at the resonance center, the magnitude and position of which can be flexibly controlled by the graphene chemical potential, the thickness of the intermediate cavity, and the Bragg grating period number. By optimizing the layer spacing and grating period, a displacement as high as 1766*λ* was achieved. Further scanning of the incident angle can maintain a high GH shift in the 18–35 µm wavelength band. This work provides a feasible solution for applications in integrated photonic devices.

## Figures and Tables

**Table 1:** Basic parameters.

Symbol	Physical Meaning	Value
*μ*	graphene chemical potential	0.3 eV
*T_g_*	environment temperature	300 K
*d_g_*	equivalent thickness of graphene layer	0.34 nm
*τ*	relaxation time	0.5 ps
*n*	refractive Indices	1.449
*θ*	incident angle	10°
*N*	number of Periods	25

**Table 2:** Changes in GH.

Controlled Parameter	Variation Range	GH Peak (λ)	Sensitivity ∂(GH)/∂param.
*μ*	0.15 → 0.5 eV	−44 → 191	583 λ/eV
*N*	5 → 35	1505 → 120	−46.2 λ/period
*τ*	0.3 → 0.5 ps	198 → 225	135 λ/ps
*n*_b_	1.36 → 1.43	159 → 157	−29 λ/RIU
*θ*	5° → 15°	98 → 398	30 λ/degree
*d*_2_	2 → 6 µm	1766 → 307	−365 λ/µm

## Data Availability

The data presented in this study are available on request from the corresponding author.

## References

[1] Berman P.R. (2002). Goos-Hänchen Shift in Negatively Refractive Media. Phys. Rev. E.

[2] Ni H., Xu S., Liu F., Xie Z., Yang J., Wang G. (2025). Goos-Hänchen shifts around Fano resonances in superconducting photonic crystals embedded with graphene. Opt. Laser Technol..

[3] Grosche S., Ornigotti M., Szameit A. (2015). Goos-Hänchen and Imbert–Fedorov shifts for Gaussian beams impinging on a hyperbolic metamaterial. Opt. Express.

[4] Wang X., Yin C., Sun J., Li H., Wang Y., Ran M., Cao Z. (2013). High-sensitivity temperature sensor using the ultrahigh-order mode-enhanced Goos-Hänchen effect. Opt. Express.

[5] Sreekanth K.V., Ouyang Q., Sreejith S., Zeng S., Lishu W., Ilker E., Dong W., ElKabbash M., Ting Y., Lim C.T. (2019). Phase-change-material-based low-loss visible-frequency hyperbolic metamaterials for ultrasensitive label-free biosensing. Adv. Opt. Mater..

[6] Lang Y., Liu Q., Wang Q., Zhou X., Jia G. (2022). Wavelength-dependent Goos-Hänchen shifts observed in one-dimensional photonic crystal films with different structures. Phys. Lett. A.

[7] Huang Y., Zhao B., Gao L. (2012). Goos-Hänchen shift of the reflected wave through an anisotropic metamaterial containing metal/dielectric nanocomposites. J. Opt. Soc. Am. A.

[8] Zheng Z., Lu F., Jiang L., Jin X., Dai X., Xiang Y. (2019). Enhanced and controllable Goos-Hänchen shift with graphene surface plasmon in the terahertz regime. Opt. Commun..

[9] Ahmad A., Haneef M., Khan H., Ahmad S., Dahshan A. (2022). The Goos-Hänchen shifts in the reflection/transmission beams under Kerr nonlinearity, Doppler broadening and Compton scattering. Opt. Laser Technol..

[10] Felbacq D., Moreau A., Smaâli R. (2003). Goos-Hänchen effect in the gaps of photonic crystals. Opt. Lett..

[11] Luo C., Guo J., Wang Q., Xiang Y., Wen S. (2013). Electrically controlled Goos-Hänchen shift of a light beam reflected from the metal-insulator-semiconductor structure. Opt. Express.

[12] Zhang Y., Wu J., Zhao L., Wang P. (2013). Goos-Hänchen shifts of a light beam reflected from the interface of colloidal ferrofluids. Optik.

[13] Zhou X., Tang P., Yang C., Liu Z. (2021). Temperature-dependent Goos-Hänchen shifts in a symmetrical graphene-cladding waveguide. Results Phys..

[14] Li Z., Zhang C., Hong Y., Da H., Yan X. (2022). Enhanced Goos-Hänchen shift of graphene via hybrid structure with dielectric grating, metallic layer and photonic crystal. Phys. E Low-Dimens. Syst. Nanostructures.

[15] Yang J., Xie Z., Ni H., Chen X., Qin Z., Zhao D., Zhao M. (2025). Giant spatial Goos-Hänchen shift achieved in superconducting hyperbolic metamaterials with graphene. Opt. Express.

[16] Cui L., Wang J., Sun M. (2021). Graphene plasmon for optoelectronics. Rev. Phys..

[17] Yang X., Liao Z., Chu X., Da H. (2023). Enhanced Goos-Hänchen shift in a defective Pell quasiperiodic photonic crystal with monolayer MoS_2_. Appl. Opt..

[18] Khan A., Mahmoud E.E., Ahmad I., Ali T. (2023). Topological localized region of Goos-Hänchen shifts in reflection and transmission. Results Phys..

[19] Ullah R., Khan S., Amina, Khan J. (2024). Tunable cratering of lateral Goos-Hänchen shifts in reflection and transmission of structured light in chiral atomic medium. Eur. Phys. J. Plus.

[20] Chang K., Li Z., Gu Y., Liu K., Chen K. (2022). Graphene-integrated waveguides: Properties, preparation, and applications. Nano Res..

[21] Chen X., Dong J., Zhao D., Ni H., Liu F., Wu H., Zhao M., Yang J. (2025). Dynamically tunable photonic manipulation and sensing in graphene array-driven Bragg gratings. iScience.

[22] Rahnama A., Hnatovsky C., Lausten R., Walker R.B., De Silva K., Mihailov S.J. (2025). Highly efficient fiber Bragg grating spectrometer fabricated with violet and near-infrared femtosecond laser pulses and the phase mask technique. Opt. Lett..

[23] Rao Y.-J. (1997). In-Fibre Bragg Grating Sensors. Meas. Sci. Technol..

[24] Wu Y., Yao B., Zhang A., Rao Y., Wang Z., Cheng Y., Gong Y., Zhang W., Chen Y., Chiang K.S. (2014). Graphene-coated microfiber Bragg grating for high-sensitivity gas sensing. Opt. Lett..

[25] He X., Liu Z.B., Wang D.N. (2012). Wavelength-tunable, passively mode-locked fiber laser based on graphene and chirped fiber Bragg grating. Opt. Lett..

[26] Ma T., Yuan J., Wang F., Liu H., Zhou X., Long K., Yu C., Liu Y. (2020). Graphene-coated two-layer dielectric loaded surface plasmon polariton rib waveguide with ultra-long propagation length and ultra-high electro-optic wavelength tuning. IEEE Access.

[27] Zhao D., Wang L., Liu F., Zhong D., Wu M. (2021). Photonic stopband filters based on graphene-pair arrays. Appl. Sci..

[28] Wang Z., Bing W., Hua L., Wang J., Lou C. (2015). Plasmonic lattice solitons in nonlinear graphene sheet arrays. Opt. Express.

[29] Liu A., Zhao D., Dong J., Zhao M., Zhong D., Ni H., Liu F., Chen X. (2025). Optical multi-wavelength selectors based on distributed feedback chirped grating in arrays of graphene. Sci. Rep..

[30] Qin C., Wang B., Huang H., Long H., Wang K., Lu P. (2014). Low-loss plasmonic supermodes in graphene multilayers. Opt. Express.

[31] Kaviani H., Barvestani J. (2022). Photonic crystal-based biosensor with the irregular defect for detection of blood plasma. Appl. Surf. Sci..

[32] Artmann K. (1948). Berechnung der Seitenversetzung des totalreflektierten Strahles. Ann. Phys..

[33] Yan Y., Zha M., Liu J., Tu J., Liu Z. (2024). Tunable giant Goos-Hänchen shift in Au-ReS_2_-graphene heterostructure. Opt. Lett..

[34] Li X., Wang P., Xing F., Chen X.-D., Liu Z.-B., Tian J.-G. (2014). Experimental observation of a giant Goos–Hänchen shift in graphene using a beam splitter scanning method. Opt. Lett..

[35] Ismach A., Druzgalski C., Penwell S., Schwartzberg A., Zheng M., Javey A., Bokor J., Zhang Y. (2010). Direct chemical vapor deposition of graphene on dielectric surfaces. Nano Lett..

[36] Liu J., Park S., Nowak D., Tian M., Wu Y., Long H., Wang K., Wang B., Lu P. (2018). Near-field characterization of graphene plasmons by photo-induced force microscopy. Laser Photonics Rev..

[37] Faneca J., Trimby L., Zeimpekis I., Delaney M., Hewak D.W., Gardes F.Y., Wright C.D., Baldycheva A. (2020). On-chip sub-wavelength Bragg grating design based on novel low loss phase-change materials. Opt. Express.

[38] Sun B., Wei M., Lei K., Chen Z., Sun C., Li J., Li L., Lin H. (2023). Integrated Bragg grating filters based on silicon–Sb_2_Se_3_ with non-volatile bandgap engineering capability. Opt. Express.

[39] Yao J., Zhang W. (2020). Fully reconfigurable waveguide Bragg gratings for programmable photonic signal processing. J. Light. Technol..

[40] Burla M., Li M., Cortés L.R., Wang X., Fernández-Ruiz M.R., Chrostowski L., Azaña J. (2014). Terahertz-bandwidth photonic fractional Hilbert transformer based on a phase-shifted waveguide Bragg grating on silicon. Opt. Lett..

[41] Zhao D., Ke S., Liu Q., Wang B., Lu P. (2018). Giant Goos-Hänchen shifts in non-Hermitian dielectric multilayers incorporated with graphene. Opt. Express.

[42] Li T., Da H., Du X., He J., Yan X. (2020). Giant enhancement of Goos-Hänchen shift in graphene-based dielectric grating. J. Phys. D Appl. Phys..

